# Cortical proteins may provide motor resilience in older adults

**DOI:** 10.1038/s41598-021-90859-3

**Published:** 2021-05-28

**Authors:** Aron S. Buchman, Lei Yu, Shahram Oveisgharan, Vladislav A. Petyuk, Shinya Tasaki, Chris Gaiteri, Robert S. Wilson, Francine Grodstein, Julie A. Schneider, Hans-Ulrich Klein, Philip L. De Jager, David A. Bennett

**Affiliations:** 1 Rush Alzheimer’s Disease Center, Rush University Medical Center, 1750 West Harrison Street, Jelke Building, Suite 100, Chicago, IL 60612 USA; 2 Department of Neurological Sciences, Rush University Medical Center, Chicago, IL USA; 3 Biological Sciences Division, Pacific Northwest National Laboratory, Richland, Washington USA; 4 Department of Psychiatry and Behavioral Sciences, Rush University Medical Center, Chicago, IL USA; 5 Department of Internal Medicine, Rush University Medical Center, Chicago, IL USA; 6 Department of Pathology, Rush University Medical Center, Chicago, IL USA; 7 Center for Translational & Computational Neuroimmunology, Department of Neurology, Columbia University Medical Center, New York, NY USA

**Keywords:** Risk factors, Neurodegeneration, Neural ageing

## Abstract

Motor resilience proteins may be a high value therapeutic target that offset the negative effects of pathologies on motor function. This study sought to identify cortical proteins associated with motor decline unexplained by brain pathologies that provide motor resilience. We studied 1226 older decedents with annual motor testing, postmortem brain pathologies and quantified 226 proteotypic peptides in prefrontal cortex. Twenty peptides remained associated with motor decline in models controlling for ten brain pathologies (FDR < 0.05). Higher levels of nine peptides and lower levels of eleven peptides were related to slower decline. A higher motor resilience protein score based on averaging the levels of all 20 peptides was related to slower motor decline, less severe parkinsonism and lower odds of mobility disability before death. Cortical proteins may provide motor resilience. Targeting these proteins in further drug discovery may yield novel interventions to maintain motor function in old age.

## Introduction

Age-related motor decline is heterogeneous with some adults showing little loss of function and others showing rapid loss of function. In prior work, we found that while nearly all older adults show some degree of Alzheimer’s disease and other brain pathologies, the extent to which these pathologies contribute to motor impairment varies widely from person to person^1,2^. This suggests that other factors, such as lifestyles or proteins may offset the negative effects of brain pathologies via other molecular pathways.

Structural (e.g., cytoskeleton, channel) and effector (e.g., signaling, enzymes) proteins are the physical bases of neural networks linking risk factors with motor decline. Neurodegenerative pathologies are associated with misfolded or abnormally activated proteins which drive the negative effects of accumulating brain pathologies on motor function. Other unidentified proteins, unrelated to the presence of brain pathologies, also contribute to motor decline. Identifying these latter proteins is crucial as they may provide motor resilience i.e. offset the negative effects of accumulating brain pathologies that contribute to motor decline. Resilience may be a high value therapeutic target that could offset the motor effects of many combinations of brain pathologies, nearly all of which are currently untreatable^3^. Few studies have identified cortical proteins that provide motor resilience^4^.

**Figure 1:**
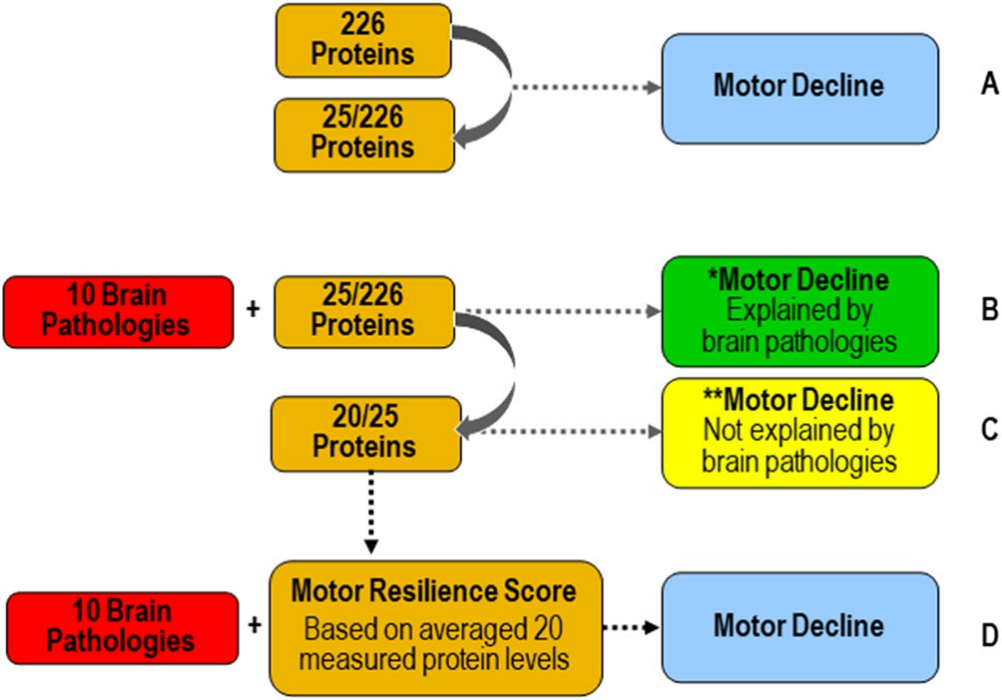
Approach for identifying cortical proteins associated with motor decline unexplained by brain pathologies. Motor decline can be partitioned in to two components. Some but not all of motor decline is explained by the negative effects of brain pathologies (green box) and some is not explained by brain pathologies (yellow box). Cortical proteins associated with motor decline not explained by brain pathologies may provide motor resilience. First, we identified cortical proteins which were associated with motor decline. We used linear-mixed effect model to examine the association between 226 proteins, measured in the dorsal lateral prefrontal cortex, with the rate of motor decline, controlling for age and sex. There were 25 proteins associated with motor decline after false discovery rate correction (**A**). Some portion of motor decline is due to the presence of brain pathologies. Therefore, we added terms for 10 indices of brain pathologies to the models of the 25 proteins associated with motor decline to regress out motor decline related to brain pathologies. Five of 25 proteins were no longer associated with motor decline after adding terms for brain pathologies (**B**). Twenty of 25 proteins remained associated with motor decline not explained by brain pathologies after correction for false discovery rate (**C**). These 20 proteins may provide motor resilience and offset the deleterious motor effects of brain pathologies which commonly accumulate in aging brains. We hypothesized that an individual’s overall resilience might be the balance of the expression levels of all of these proteins. So, we averaged the levels of all 20 proteins to construct a person-specific motor resilience score. We then examined the association of the motor resilience protein score in a linear mixed-effect model including terms for 10 indices of brain pathologies with motor decline when controlling for age and sex (**D**).

In prior work we identified cognitive resilience proteins by adding terms to control for indices of brain pathologies to models examining cognitive decline to isolate cognitive decline unexplained by brain pathologies. Proteins related to cognitive decline unexplained by brain pathologies may provide cognitive resilience^5,6^. This study extends this approach (Fig. 1) to identify motor resilience proteins by using data from older autopsied decedents from two cohort studies with longitudinal motor function over many years prior to death with adequate measures of postmortem brain pathologies in whom cortical proteins that might provide motor resilience were measured^7^.

By controlling for indices of ten brain pathologies, while modeling motor decline, we identified 20 cortical proteotypic peptides measured in the prefrontal cortex from more than 1200 well-characterized older adults associated with motor decline unexplained by brain pathologies i.e., motor resilience. More of some peptides and less of others offset the negative effect of multiple brain pathologies on motor decline. We aggregated the expression levels of these peptides to yield a person-specific motor resilience protein score that might predict motor resilience. Supporting this notion, a higher motor resilience protein score was associated with slower motor decline, less severe parkinsonism and disabilities proximate to death. These findings suggest that all individuals in these analyses manifest some degree of motor resilience with some having higher than average and some having lower than average resilience. Targeting these proteins in further drug discovery offers the potential for novel therapeutics that could modulate these proteins and their molecular pathways to maintain motor function in aging adults even in the absence of treatments for Alzheimer's disease and other brain pathologies.

## Results

### Motor decline in the analytic cohort

**Figure 2:**
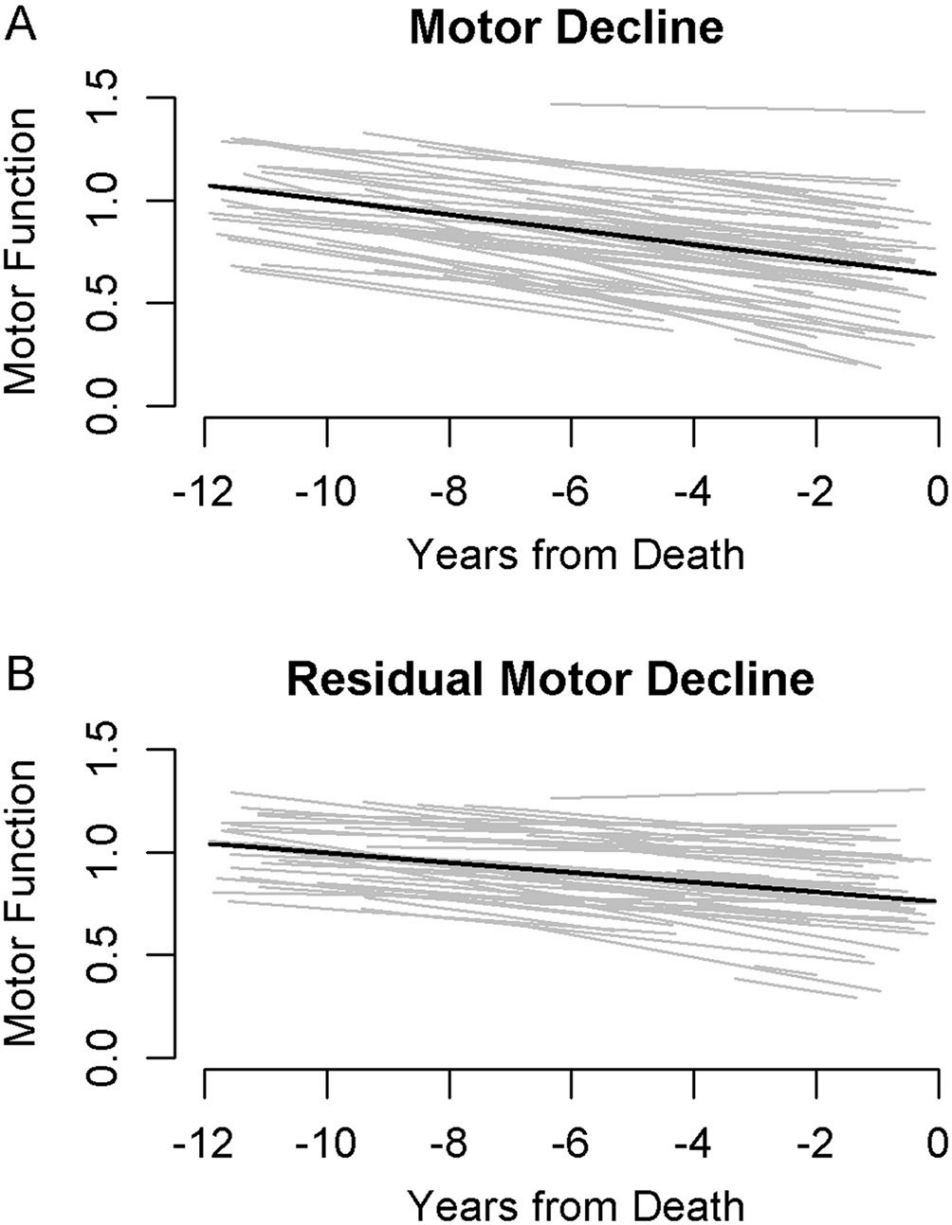
Heterogeneity of motor decline with and without controlling for Alzheimer's disease and other brain pathologies. We randomly selected a subset of 71 individuals included in the analyses of the full cohort (N = 1226) to illustrate the heterogeneity of motor decline without adjustment for Alzheimer's disease and other brain pathologies (2A) and for residual motor decline after including terms in the same model which adjusted for indices of ten Alzheimer’s disease and related brain pathologies (2B). (**A**) Trajectories of motor decline based on repeated annual measures of motor testing prior to death (Fig. 1, Model 1a). Each individual light line represents the estimated person-specific decline for a single adult with the length of the line based on the number of years of follow-up. Bold black line represents average motor decline (Estimate slope = − 0.036 standardized unit/year). (**B**) Trajectories of residual motor decline in the same individuals shown in Fig. 2a to illustrate the heterogeneity of residual motor decline after adding terms to the models in 2a for ten indices of brain pathologies. Light lines show person-specific residual motor decline and bold black line represents average residual motor decline (Estimate slope = -0.024 standardized unit/year).

There were 1226 adults included in these analyses and their clinical characteristics at their last visit proximate to death and autopsy findings are summarized in Table 1. On average, global motor function declined about 0.04 unit/yr (Standard Error: 0.001, p < 0.001). To allow visualization of person-specific slopes we chose a random sample of 71 individuals from the full analytic cohort included in the prior model. Figure 2A shows that there was much heterogeneity of motor decline with many individuals showing slower or faster than average motor decline.

**Table 1:** Clinical and postmortem characteristic of the analytic cohort.

Measure	Mean (SD) or number (%)
**Clinical measures at last visit**	
Age at death (years)	89. 3 (6.51)
Females (%)	811 (68.0%)
Education (years)	16.2 (3.59)
Purdue pegboard test (no. of pegs/30 s)	6.7 (3.37)
Finger-tapping test (taps/10 s)	47.2 (14.57)
Time to cover a distance of 8 feet (seconds)	6.0 (4.23)
Number of steps required to cover 8 feet (steps)	9.0 (3.42)
360 degree turn time (seconds)	7.9 (6.33)
Number of steps to complete a 360° turn (steps)	12.4 (5.69)
Leg stand (seconds)	2.18 (2.77)
Toe stand (seconds)	3.03 (3.54)
Grip strength (kilograms)	32.4 (18.17)
Pinch strength (kilograms)	8.14 (5.00)
Global motor measure	0.67 (0.26)
Global parkinsonism score	17.3 (11.58)
Instrumental activities of daily living disability	1076 (90.3%)
Activity of daily living disability	757 (63.5)
Mobility disability	1038 (87.1)
**Postmortem Indices**	
Postmortem interval (hours)	8.4 (6.04)
Nigral neuronal loss (moderate-severe)	155 (13%)
Lewy bodies (any)	316 (27%)
Pathologic Alzheimer's disease (NIA Reagan)	766 (64%)
TDP-43 (present beyond amygdala)	380 (32%)
Hippocampal Sclerosis (present)	113 (9%)
Macroinfarcts present	430 (36%)
Microinfarcts present	337 (28%)
Atherosclerosis (moderate-severe)	405(34%)
Arteriolosclerosis (moderate-severe)	397 (33%)
Cerebral amyloid angiopathy (moderate-severe)	422 (35%)

### Brain pathologies and motor decline

**Figure 3:**
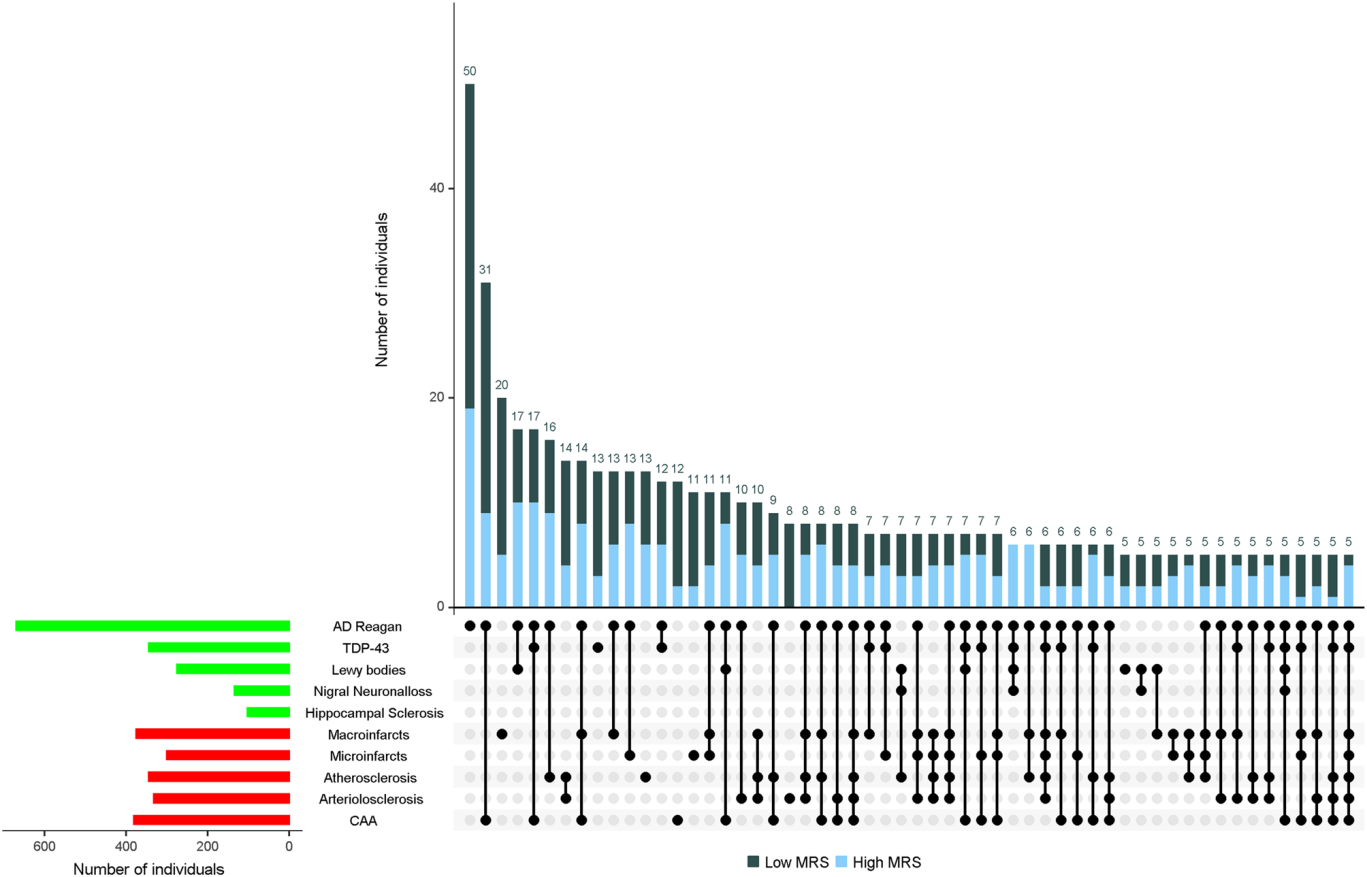
Motor resilience proteins mitigate the effects of heterogenous combinations of Alzheimer's disease and other brain pathologies in older brains. The bar chart in the lower left corner shows the frequencies of ten individual brain pathologies indices collected in this study. One or more pathologies were observed in almost 95% of decedents. Connected black dots on the x-axis indicate the specific combination of brain pathology in 5 or more individuals. The second bar chart in the main panel show the frequencies of the brain pathology indices for persons with low or high motor resilience protein stratified by the median value for motor resilience protein [high motor resilience protein (blue) versus low motor resilience protein (black)], ordered by their frequency. The height of each bar corresponds to the number of persons with each combination. *AD* Alzheimer’s disease pathology; *CAA* cerebral amyloid angiopathy. As illustrated in the figure, brain pathology indices frequently co-occur. More than 80% of older adults in these analyses showed combinations of two or more brain pathologies.

For descriptive purposes, we dichotomized the ten brain pathologies measured as noted in Table 1 to count how many pathologies were observed in each individual examined. A typical individual had 3 of the 10 pathologies (range 0–9, median 3; Q1–2, Q3–4). As illustrated in Fig. 3 by the blue bar on the bottom left of the figure, we did not observe any of the ten pathologies in about 5% of individuals; one or more pathologies were observed in nearly all persons (95%) and more than 80% showed combinations of 2 or more brain pathologies.

In a single linear mixed-effects model we examined the association of the 10 brain pathologies and demographics with level of motor function proximate to death and to the annual rate of change in motor decline. This model included 25 terms including Time, the annual rate of change in motor decline prior to death, as well as age, sex, and 10 indices of brain pathologies and their interactions with Time.

This single model showed that Alzheimer's disease pathology (Estimate, − 0.004, S.E., 0.002, p-Value, 0.009), nigral neuronal loss (Estimate, − 0.004, S.E., 0.001, p-Value, 0.002), macroinfarcts (Estimate, − 0.004, S.E., 0.002, p-Value, 0.034), and atherosclerosis (Estimate, − 0.003, S.E., 0.001, p-Value, 0.004), were associated with the annual rate of motor decline (Supplementary eTable 2).

To visualize the heterogeneity of motor resilience, Fig. 2B shows the different trajectories of motor decline after adding terms to our models that control for indices of ten brain Alzheimer’s disease and related pathologies for the same subset of 71 individuals shown in Fig. 2A. The heterogeneity of motor decline can be observed in both figures. The variance of person-specific slopes of motor decline (light lines) after controlling for Alzheimer’s disease and related pathologies in Fig. 2B is 6.8% less as compared to the person-specific variance of slopes for motor decline in Fig. 2A. The reduction in the variance of person-specific slopes of decline after addition of brain pathologies indicate that part of motor decline was due to the burden of brain pathologies, yet additional factors or proteins that are independent of brain pathologies and drive motor resilience remain to be identified.

### Cortical proteins and motor decline

We examined 226 peptides corresponding to 126 proteins in separate linear mixed-effects models that controlled for age at death and sex. For 82 of 126 proteins, we employed 2 or more peptides to ensure the fidelity of expression levels that were measured (Supplementary eTable 1). For 90% (74/82) of the proteins for which measured multiple peptides, both peptides showed similar associations with motor decline (Supplementary eTable 1). Ten percent (8/82) showed one peptide which was associated with motor decline and the second peptide which did not survive the false discovery rate correction (Supplementary eTable 1). Twenty-five of 226 peptides that were associated with the rate of motor decline and survived false discovery rate adjusted p < 0.05 were retained for the next stage of our analysis (Fig. 1A).

Next to identify proteins associated with motor decline that were not explained by brain pathologies i.e., motor resilience, we repeated a separate linear mixed-effect models for each of the 25 proteotypic peptides associated with motor decline adding terms for 10 indices of brain pathologies (Fig. 1B).

**Figure 4:**
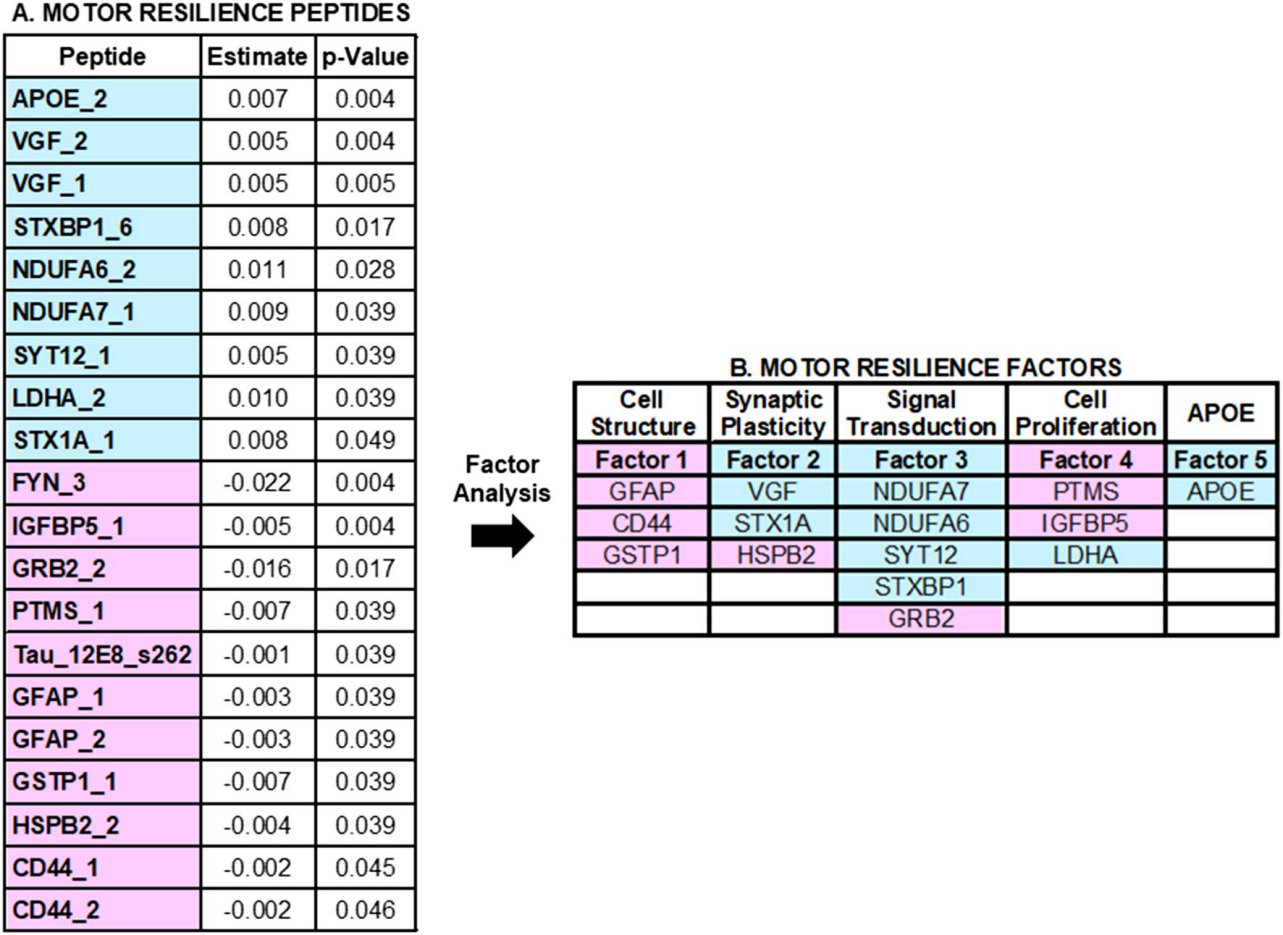
Cortical Proteotypic Peptides and Residual Motor Decline. (**A**) Shows the 20 proteotypic peptides which were associated with the annual rate of residual motor decline. Each row in Table A shows the results of a separate linear mixed-effect model that included terms for a specific peptide, demographics and 10 indices of brain pathologies. Each row shows the strength of the association [Estimate and corrected p-Value (false discovery rate 5%)] of a single peptide with the annual rate of motor decline (Time*peptide). Peptides are ordered by those for which a higher level was associated with slower motor decline (blue) and those for which a lower level was associated with slower motor decline (pink) and by strength of their association with motor decline). (**B**) shows the clustering of the peptides into five factors. Each factor may represent a distinct biological function.

**Figure 5:**
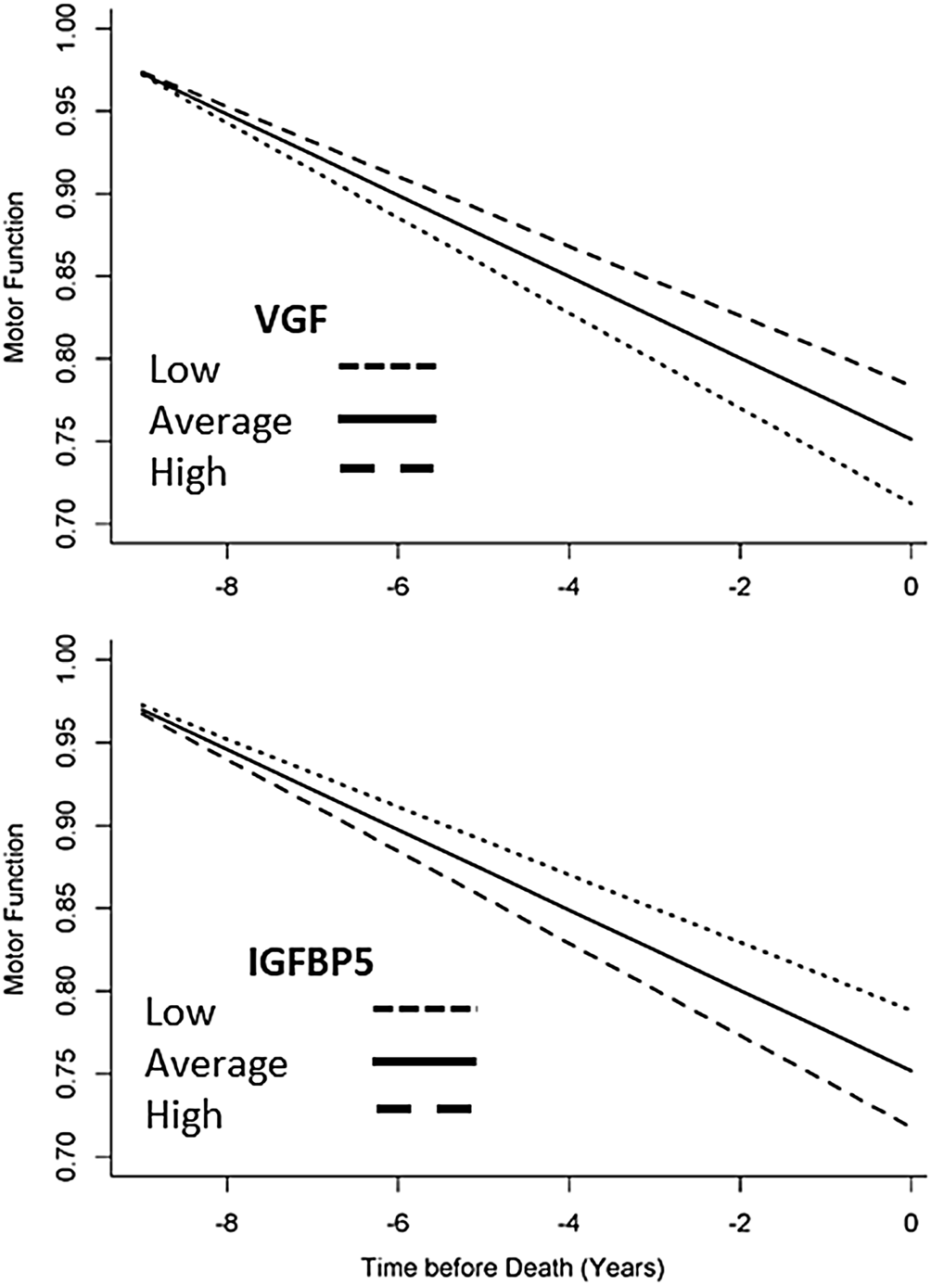
Motor resilience proteins are related of the annual rate of motor decline prior to death in older adults. Figures show model generated trajectories for motor decline for three average participants with low (10th percentile), average (50th percentile) or high (90th percentile) expression levels for VGF or IGFBP5. A higher level of VGF is associated with slower motor decline and a lower level of IGFBP5 is associated with slower motor decline.

Twenty of 25 peptides (Fig. 1C) remained associated with motor decline unexplained by brain pathologies (false discovery adjusted p < 0.05) and are listed in Fig. 4A. Higher levels of nine peptides were associated with slower motor decline (i.e., a positive sign for the beta of the association of the protein with motor decline (Fig. 4A, blue). Lower levels of eleven peptides were associated with slower motor decline (i.e., a negative sign for the beta of the association of the peptides with motor decline (Fig. 4A, red). Figure 5 illustrates the associations for the two peptides most strongly associated with slower motor decline.

### Motor resilience protein score and motor decline

As higher and lower levels of some proteotypic peptides are related to slower motor decline, we hypothesized that the degree of an individual’s resilience might depend on the balance of the expression levels of all twenty peptides associated with motor decline. To test this hypothesis, we calculated a motor resilience protein score for each person by weighting and averaging the person-specific expression levels for each of the 20 peptides and multiplying the expression level by the estimate for each peptide’s interaction with annual rate of motor decline after controlling for brain pathologies (Fig. 4A, Estimates).

We examined the association of the motor resilience score with motor decline in a model controlling for demographics and ten brain pathologies (Fig. 1D). As hypothesized, motor resilience protein score was associated with the rate of motor decline (motor resilience protein score, Estimate, 0.006, S.E., 0.0009, p < 0.001); a higher-than-average score was associated with slower motor decline and a lower-than-average motor resilience score with a faster rate of motor decline.

**Figure 6:**
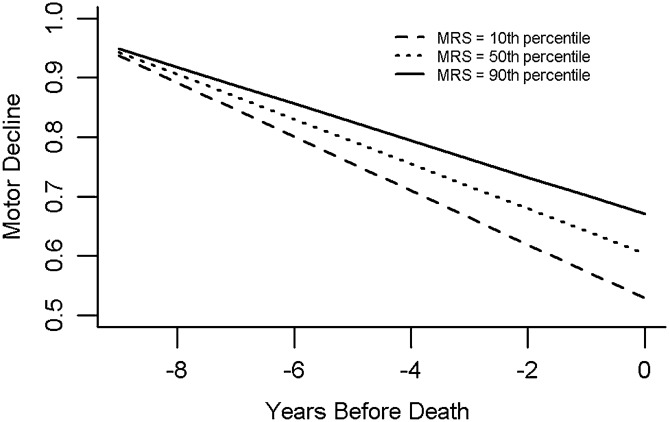
Motor resilience protein score is related to the annual rate of motor decline. Person-specific resilience is the overall balance of all peptides which drive residual motor decline. We calculated a person-specific motor resilience protein score based on the expression levels of the 20 proteotypic peptides associated with motor decline (Table 2A). The figure illustrates the model derived trajectory of motor decline for three average older adults with high (motor resilience protein 90th percentile: decline = − 0.031/year), average (motor resilience protein score 50th percentile: decline = − 0.038/year) and low (motor resilience protein score10th percentile: decline = − 0.045/year) motor resilience protein score.

Figure 6 illustrates the trajectories of motor decline for three similar individuals with high, average and low motor resilience score. This figure shows that an average female participant, 89 years old at the time of death with a high motor resilience protein score (90^th^ percentile) had a 70% slower annual rate of motor decline compared to an individual with a low motor resilience protein score (10th percentile).

Both terms for motor resilience protein score and age were related to the rate of motor decline in the previous model. By inspecting the terms of this model, we can contextualize the effect of the motor resilience protein score metric by comparing the estimate of its association with motor decline (prior paragraph) to the estimate of the association of a more familiar metric, age with motor decline which is included in the same model (Age, Estimate, 0.0003, S.E., 0.0001, p = 0.016). A 1-SD increase in motor resilience protein score was equivalent to the slower rate of motor decline associated with being 20 years younger at the time of death (Estimate *motor resilience protein score*, 0.006/Estimate *Age*, 0.0003 = 20).

Compared to a model which included only terms for age and sex, motor resilience score accounted for 9.7% additional variance of motor decline. The ten indices of brain pathology treated as a single block accounted for an additional 6.8% of additional variance of motor decline.

### Motor resilience protein score and other motor aging phenotypes

We next tested whether individuals with higher scores manifested less severe deficits in other aging motor phenotypes. In support of the construct validity of the motor resilience protein score, higher level of motor resilience protein score was associated with less severe parkinsonism (Estimate − 0.255, S.E., 0.041, p < 0.001) and lower odds of severe disabilities including instrumental activities of daily living disability (OR 0.63, 95% CI 0.56, 0.70), activities of daily living disability (OR 0.67, 95% CI 0.60, 0.74) and mobility disability (OR 0.87, 95% CI 0.77, 0.97) proximate to death.

### Cortical proteins and motor resilience factors

An exploratory factor analysis was performed to examine if these 20 peptides might cluster into factors that share common physiologic functions to provide resilience. We applied factor analysis to the 20 peptides. Five factors with eigenvalues above 1.0 accounted for about 69% of the total variance, and peptides with factor loadings > 0.5 after varimax rotation were retained for each factor (Supplementary eTable 3). The peptides for each factor are shown in Fig. 4B and details about each of the factors potential functions are discussed in Supplementary eTable 3. We averaged the measured levels of each of the peptides within each factor and all five factors were associated with motor decline (Table 2 and Supplementary Fig. 1).

**Table 2:** Motor resilience factors and motor decline.

Factor	Function	Level of motor function estimate (S.E., p-Value)	Motor decline * factor estimate (S.E., p-Value)
Factor 1	Cell structure	0.029 (0.009, < 0.001)	0.003 (0.001, < 0.001)
Factor 2	Synaptic plasticity	0.044 (0.009, < 0.001)	0.004 (0.001, < 0.001)
Factor 3	Signal transduction	0.042 (0.009, < 0.001)	0.005 (0.001, < 0.001)
Factor 4	Cell proliferation	0.049 (0.008, < 0.001)	0.005 (0.001, < 0.001)
Factor 5	APOE	0.025 (0.008,0.002)	0.002 (0.0009,0.007)

The exploratory factor analysis applied to the proteotypic peptides identified in the current study (Fig. 4) identified five different factors. Inspection of the peptides clustering within each factor suggests that each factor may represent a distinct biological function. We constructed an average score for each of the 4 factors based on the constituent peptides obtained for each factor. There was only one protein in Factor 5. Each row shows the results for a single linear mixed-effect model which examines the association of the level of each of the 5 factors with the level of global motor score proximate to death and the interaction of each factor with the annual rate of change in global motor score prior to death (Motor Decline * Factor). Each cell shows the estimate (standard error and p-value) for the term shown in the left-hand column. Each model also included an additional 5 terms including Time (annual rate of motor decline), Age, Sex and their interaction with the annual rate of motor decline which are not shown.

## Discussion

In prior work, we identified two molecular pathways related to cognitive decline in aging adults. Some proteins drive cognitive decline via brain pathologies like β-amyloid and some proteins that drive cognitive decline unexplained by brain pathologies provide cognitive resilience^6^. Little prior research has examined motor decline unexplained by brain pathologies because of the inherent challenges, including the need for longitudinal motor function over many years prior to death, and adequate measures of pathologies and resilience markers^4^. Studies restricted to clinical and biomarker data can only account for some underlying pathologies (e.g., macroinfarcts, cerebrospinal fluid amyloid and tau, amyloid positron emission tomography) and can only obtain indirect measures of resilience (e.g., fluoro-D-glucose positron emission tomography). Other pathologies (e.g., microinfarcts, TAR DNA-binding protein-43) and resilience markers can only be studied in post-mortem brain tissue. This study analyzed novel selected reaction monitoring proteomic data that had previously been collected from the prefrontal cortex, a crucial brain region for translating the goal of a volitional movement, its motor planning and execution in a large number of community-dwelling older adults^8^. Building on our findings in the cognitive systems the current study shows that by probing motor decline unexplained by brain pathologies i.e., motor resilience, we were able to discover novel cortical proteins which may provide motor resilience.

Adults differ in their ability to tolerate accumulating brain pathologies, which is called resilience or neural reserve^3,9^. In addition to structural redundancies, the brain is plastic, actively responding to damage, behavior and past experiences. Resilience includes both functional compensation through engagement of redundant neuronal populations and dynamic genes and peptides which provide molecular resilience to maintain cellular homeostasis to counteract senescence. Some limit the concept of resilience to factors which have a beneficial effect on an individual’s ability to tolerate pathology^9^. The conceptualization used in this study assumes that all living brains have some degree of resilience which is the balance between many proteins and factors some which may increase and some which decrease brain resilience^3^.

To lend empiric support for this expanded conceptualization of motor resilience, we aggregated the measured levels of twenty cortical proteins associated with motor decline to calculate a person-specific motor resilience score. A higher score was associated with slower motor decline and less severe motor impairments and disabilities prior to death, suggesting that everyone has some degree of resilience. These data support the notion, that all adults may have some degree of resilience^10,11^; adults with rapid motor decline may have lower than average resilience, while those manifesting slower than average motor decline may have high resilience.

There are many gaps in our knowledge about the potential motor resilience proteins discovered in this study. First, the associations of these proteins with motor decline need to be replicated in further studies. Assuming that these associations are replicated, additional studies with other aging phenotypes are needed to determine the specificity of resilience proteins identified in this study and if they may provide resilience for other aging phenotypes. The functional mechanisms underlying these resilience proteins are not known and will require further experiments. The exploratory factor analysis applied to the proteotypic peptides identified in the current study identified five different factors (Fig. 4B). Inspection of the peptides clustering within each factor suggests that each factor may represent a distinct biological function (Supplementary Table 3) for example cell structure integrity^12–14^, synaptic plasticity^15–20^, mitochondrial bioenergetics, signal transduction and cell proliferation^4,21–27^ and amyloid^28^.

The current results also provide novel data about the basis for late-life motor impairment. Recent work examining diverse motor phenotypes has suggested that while a higher burden of mixed-brain pathologies contribute to poorer motor function, these indices account for only a minority of motor decline^2^. Consistent with these findings, compared to a model which included only terms for age and sex, the ten indices of brain pathology accounted for an additional 7% of additional variance of motor decline. In contrast, in these same models, the motor resilience peptides identified accounted for an additional 10% of motor decline which was unexplained by brain pathologies. Thus, identifying peptides associated with residual motor decline offers the potential for a more complete understanding of the diverse mechanisms underlying motor decline that may not manifest with an observable pathologic footprint.

When it is feasible it is common to examine several peptides to ensure the fidelity of the expression levels which are being measured. In the current study, we examined most proteins with two peptides. For about 90% of the proteins, both peptides showed similar associations with motor decline i.e., both were either related or unrelated to motor decline. A small number showed a divergence of associations between both peptides and motor decline.

There are several possible reasons for these divergences. One possible cause may be due to the presence of posttranslational modifications in the regions covered by the peptides. For example, Apolipoprotein E protein was measured with two peptides covering regions 138–152 and 210–224. The correlation between the peptides is 84%. However, the presence of serine phosphorylation^29^ and O-linked β-N-acetylglucosamine^30,31^ at the 147 and 212 residues may have subtle effects that contribute to the differences in the associations of both peptides.

Another example of Plexin B1 protein, which is measured with two peptides showing correlation of only 56%. However, one peptide (1274–1284) is mapped to the luminal part of the receptor, while the one (1596–1613) matches the transmembrane region separated by a known cleavage site^32^. The cleavage site at the location 1305/1306 may account for the differences in the associations of both peptide’s with motor decline, as the two resulting protein domains may have different fates. The amyloid precursor protein is a classic example. It undergoes cleavage by beta-site amyloid precursor protein cleaving enzyme 1 and gamma-secretase enzymes. Each protein fragment has its own fate and only the one located between the cleavage sites of the aforementioned proteases, amyloid beta (Aβ), is associated with cognitive decline^33,34^.

Finally, the decision to include a peptide as a potential motor resilience protein was based simply on whether it survived false discovery rate. Thus, random variability might affect one but not the second peptide. Together these observations illustrate that in most cases it is safe to infer relative protein abundance using only one peptide, but there can be exceptions that may be affected by specific protein biochemistry.

The study has a number of strengths. Findings are based on large numbers of well-characterized male and females who underwent repeated assessments of motor function with validated instruments. Autopsy rates were very high, ten brain pathologies were assessed using a structured postmortem assessment, and the statistical approach leveraged person-specific information.

Yet this study has limitations that need to be considered. These findings are from a selected cohort which will need to be replicated in population-based studies. Not all known pathologies were measured as brain white matter integrity measures were not included. Only a small number of peptides were analyzed in the current study and the peptides which were examined were chosen for their presumed associations with Alzheimer’s disease and related pathologies phenotypes. Broader-based measurement of the entire proteome using unbiased discovery may facilitate pathway and network analyses to help determine the mechanisms through which peptides unrelated to brain pathologies contribute to motor decline^5^. Third, in contrast to cognition, the motor pathways extend beyond the brain to reach muscle in the periphery. Thus, to probe motor resilience it will be necessary to extend these studies to account for both peptides and degenerative changes throughout the entire motor pathway both within other motor cortices as well as motor structures outside the brain.

## Methods

### Study participants

Participants were older persons enrolled in one of two ongoing cohort studies of chronic conditions of aging, the Religious Orders Study and the Rush Memory and Aging Project^7^. Participants entered the studies without known dementia and agreed to annual assessments as well as brain donation after death. Both cohort studies (Rush Memory and Aging Project and Religious Order Study) were approved by an Institutional Review Board of Rush University Medical Center. Written informed consent was obtained from each study participant as was an Anatomical Gift Act for organ donation. All data collection was performed in accordance with relevant guidelines and regulations.

A prospective subset of 1226 autopsied decedents underwent targeted proteomics data collected as part of the Accelerating Medicines Partnership- Alzheimer's disease consortium study from dorsolateral prefrontal cortex as described below. The dorsal lateral prefrontal cortex was chosen based on a large body of work which has identified the prefrontal cortex as a crucial region in a wide range of human behaviors, including motor function for translating ideas or goals into varied cognitive and motor behaviors. Thus, this single region has potential to inform on the biology underlying diverse facets of late life cognitive and physical decline^8,35, 36^.

### Motor assessments

We quantified ten motor tasks. (I&II) The Jamar hydraulic hand and pinch dynamometers (Lafayette Instruments, Lafayette) were employed to test bilateral grip and pinch strength. (III) Dexterity of the arms was based on the number of pegs placed in the Purdue Pegboard in thirty seconds. Two trials for each hand were averaged to provide a Purdue Pegboard score. (IV) An electronic tapper (Western Psychological Services, Los Angeles, CA) was employed to determine how quickly participants were able to tap with their index finger for ten seconds. Two trials for each hand were averaged to yield a tapping score. (V-VIII) We measured the time and number of steps taken to walk eight feet and turn 360°. (IX) Participant’s stood on each leg for ten seconds to assess lower extremity strength and balance. (X) Then they were requested to stand on their toes for ten seconds and the time standing was recorded^37,38^.

Composite measures reduce floor and ceiling effects and are suitable for modeling change over time. A summary global motor score was constructed by scaling and averaging all ten tests. In prior studies, a low score of this measure was associated with diverse adverse health outcomes including survival, incident disabilities and incident cognitive impairment^38,39^.

### Brain autopsy

After death, the brain was removed and hemisected following standard procedure, as previously described^40^. The hemispheres were cut into 1 cm coronal slabs. One hemisphere was prepared for histological evaluation and the other frozen. Briefly, the fresh slabs were fixed in 4% paraformaldehyde. Tissue blocks from predetermined regions were dissected, embedded in paraffin, and cut into 6 and 20 micro sections.

Postmortem indices of five neurodegenerative and five cerebrovascular disease pathologies were systematically assessed blinded to all clinical and cognitive data^41^. For descriptive purposes the ten indices described below were dichotomized as noted in Table 1. The primary analytic severity score for each of the ten pathologies included in these analyses are described below.

### Neurodegenerative pathologies

#### Nigral neuronal loss

Dissection of diagnostic blocks included a hemisection of midbrain including substantia nigra. Nigral neuronal loss was assessed in the substantia nigra in the mid to rostral midbrain near or at the exit of the 3^rd^ nerve using hematoxylin and eosin stain and 6 micron sections using a semi-quantitative scale (0–3)^42^.

#### Lewy bodies

Seven regions (substantia nigra, anterior cingulate cortex, entorhinal cortex, amygdala, midfrontal cortex, superior or middle temporal cortex, inferior parietal cortex) were assessed for Lewy bodies using α-synuclein immunostaining (LB509; 1:150 or 1:100, Zymed Labs, Invitrogen, Carlsbad, CA, USA; and pSyn#64; 1:20,000; Wako Chemicals, Richmond, VA, USA). The presence or absence of Lewy bodies in any of the seven regions was recorded.

#### Alzheimer's disease pathology

A modified Bielschowsky silver stain was used to visualize neuritic plaques, diffuse plaques, and neurofibrillary tangles in five cortical areas (hippocampus, entorhinal, midfrontal, middle temporal, and inferior parietal). A board-certified neuropathologist, blinded to clinical data, determined the pathologic diagnosis of Alzheimer's disease based on an intermediate to high likelihood of Alzheimer's disease according to NIA Reagan criteria^43^. In addition, a summary global Alzheimer's disease pathology score was made based on the greatest density of neuritic plaques, diffuse plaques, and neurofibrillary tangles in one mm^2^^44^. TAR DNA-binding protein-43*:* TAR DNA-binding protein-43 staging (1: localized to amygdala only, 2: extended to other limbic regions, and 3: extended to neocortical regions) was determined using a phosphorylated monoclonal TAR5P-1D3 antibody (pS409/410; 1:100, Ascenion, Munich, Germany)^45^.

#### Hippocampal sclerosis

The presence of hippocampal sclerosis was identified by severe neuronal loss and gliosis on hematoxylin and eosin stain -stained sections in CA1 or subiculum^46^.

### Cerebrovascular disease pathologies

#### Infarcts

Chronic *macroinfarcts* identified during gross examination were confirmed histologically. The presence of *microinfarcts* was determined on sections in a minimum of nine regions stained with hematoxylin and eosin^47^.

#### Cerebral amyloid angiopathy

Sections in 4 neocortical regions (i.e. mid frontal, mid temporal, angular and calcarine) were immunostained for β-amyloid (4G8; 1:9000, Covance Labs, Madison, WI, USA; 6F/3DDako; 1:50, North America Inc., Carpinteria, CA, USA; and 10D5; 1:600, Elan Pharmaceuticals, San Francisco, CA, USA). Meningeal and parenchymal vessels were assessed for amyloid deposition. Individuals with moderate or severe amyloid angiopathy were identified^48^.

#### Atherosclerosis

A semi-quantitative scale (0–3) was used to assess the severity of atherosclerosis was determined by visually examining the cerebral arteries and visible branches of the circle of Willis.

#### Arteriolosclerosis

The severity of arteriolosclerosis in the small vessels of the anterior basal ganglia were assessed using a semiquantitative scale (0–3)^49^.

### Targeted selective reaction monitoring proteomics

The current study analyzes targeted proteomics data collected as part of the Accelerating Medicines Partnership- Alzheimer's disease consortium study from dorsolateral prefrontal cortex using a standard protocol to prepare samples^6^. Based on suggestions from diverse sources regarding proteins associated with Alzheimer's disease and related dementia phenotypes, we nominated 228 genes and designed corresponding 523 proteotypic peptides. After collecting the selected reaction monitoring data and evaluating quality control metrics, we retained measurements of 226 peptides corresponding to 126 proteins (Supplementary Table 1). For 82 of 126 proteins, we employed 2 or more peptides to ensure the fidelity of expression levels measures. The methods are summarized below and additional details can be found in prior publications^6,50^.

A 96 well plate format using Epmotion 5075 TMX (Eppendorf) or Liquidator96 (Rainin) was used to perform liquid handling. A denaturation buffer (8 M urea, 50 mM Tris–HCl pH 7.5, 10 mM DTT, 1 mM EDTA) was used to homogenize about 20 mg of brain tissue from each individual. After denaturation and reduction, 400 µg protein aliquots were alkylated with 40 mM iodoacetamide and digested with trypsin (1:50 w/w trypsin to protein ratio). Solid phase extraction with Strata C18-E (55 µm, 70 Å) 25 mg/well 96-well plates (Phenomenex) on positive pressure manifold CEREX96 (SPEware) was employed to clean the digested samples. Tryptic peptide concentrations were readjusted to 1 µg/µL and 30 µL aliquots were mixed with 30 µL stable heavy isotope-labeled synthetic peptides. The spiked-in heavy peptides were used as standards for relative abundance quantification.

A nanoACQUITY UPLC was coupled to TSQ Vantage MS instrument and a sample injection of 2 µL was used for each of the measurements in the LC-selected reaction monitoring experiments. Buffer A used was 0.1% FA in water and buffer B was 0.1% in 90% ACN. Peptide separations were performed by an ACQUITY UPLC BEH 1.7 µm C18 column (75 µm i.d. × 25 cm) at a flow rate 350 nL/min using gradient of 0.5% of buffer B in 0–14.5 min, 0.5–15% B in 14.5–15.0 min, 15–40% B in 15–30 min and 45–90% B in 30–32 min. Tryptic peptides were quantified based on fragmentation ion intensity ratios of endogenous (light) and spiked-in (heavy) synthetic standards using Skyline software^51^. The L/H ratios were log base 2 transformed and centered at the median. These transformed relative abundances (L/H ratios) are available from a prior study and were used in the current analyses^6^.

### Other clinical covariates

Annual uniform structured clinical testing includes a medical history. Overall follow‐up rate exceeded 95%. Demographic measures including age, sex, years of education and race were recorded at baseline interview and age of death was recorded at the time of autopsy^7^.

Parkinsonism a common motor phenotype in older adults was based on assessment of 26 items from the motor portion of the original Unified Parkinson’s Disease Rating Scale by trained nurse clinicians showing reliability with a trained movement disorders physician^52^. The scores of the 4 parkinsonian signs were averaged and a global Unified Parkinson’s Disease Rating Scale score was calculated^53^.

Disability was assessed annually via three self-report instruments. Instrumental activities of daily living were assessed using eight items adapted from the Duke Older Americans Resources and Services project Instrumental scored as 0–8^54^. Basic activities of daily living were assessed using 6 items from the Katz scale scored as 0–6^55^. Mobility disability was assessed using the Rosow-Breslau scale, which assesses three walking tasks, scored 0–3^56^. The sum of the impaired items was used in these analyses.

### Analytic plan

Our prior work suggests that several analytic steps could be used to isolate proteins related to motor decline unexplained by brain pathologies^5,6^. First, we identified cortical proteins in the human prefrontal cortex associated with motor decline (Fig. 1A). Then we added terms for ten indices of brain pathologies (Fig. 1B). Controlling for brain pathologies in older adults with longitudinal measures of motor decline, would isolate proteins related to motor decline, unexplained by brain pathologies (Fig. 1C) i.e., that may provide motor resilience which offsets the deleterious motor effects of brain pathologies.

Linear mixed-effects models which controlled for age and sex were examined to determine the person-specific rate of change in motor decline. The core model included terms for Time (annual rate motor decline), age at death, sex and their interaction with Time. We examined the association of 10 indices of brain pathologies with global motor score proximate to death and the annual rate of motor decline. To identify proteotypic peptides associated with motor decline, we augmented the core model by adding a term for each peptide and its interaction with time. Each of the 226 proteotypic peptides were examined alone in a separate model. The resulting p values were adjusted using false discovery rate to correct for multiple testing. Then, we added terms for the 10 brain pathologic indices to determine which peptides remained associated with motor decline after the adjustment for pathologies (i.e. motor resilience).

Risk scores have been employed to aggregate the additive effects of multiple genes or clinical risk factors^57,58^. We used the same approach to construct a person-specific motor resilience protein score by weighting the person-specific expression level for each of the peptides associated with annual rate of motor decline after controlling for brain pathologies. We multiplied the expression level for each of these proteins by the estimate for each peptide’s interaction with the rate of motor decline. We then averaged the scores from these peptides into a single motor resilience protein score. Finally, we z-scored the person-specific motor resilience protein scores to provide standardized scores across all decedents. To validate the person-specific motor resilience protein scores, we examined its associations with motor decline using a linear-mixed effect model as described above. Then we used regression models to examine its associations with other manifestations of late-life motor impairment including the severity of parkinsonism and odds of having disabilities proximate to death.

To examine if peptides associated with motor resilience might cluster into factors that might share common physiologic functions, we performed an exploratory factor analysis. We applied factor analysis to all motor resilience peptides identified and retained all factors which had eigenvalues of ≥ 1.00. Following varimax rotation of these factors, we included all peptides in each factor with absolute factor loading > 0.50. Statistical analyses were performed using SAS/STAT software, version 9.4 for Linux (SAS Institute Inc, Cary, NC). Statistical significance was determined at α level of 0.05 unless otherwise specified.

## Supplementary Information

Supplementary Information.

## Data Availability

Data used in this study are available through request via the RADC Research Resource Sharing Hub (https://www.radc.rush.edu/).
