# Supplementary material for: Cortical proteins may provide motor resilience in older adults
Source: Sci Rep. 2021 May 28;11:11311. doi: 10.1038/s41598-021-90859-3 (PMC8163829; doi:10.1038/s41598-021-90859-3)
Supplement: Supplementary file 1 — Supplementary Information. [file 41598_2021_90859_MOESM1_ESM.pdf]

SUPPLEMENTAL MATERIAL FOR:

Cortical proteins may provide motor resilience in older adults

Aron S. Buchman, Lei Yu, Shahram Oveisgharan, Vladislav A. Petyuk, Shinya Tasaki, Chris Gaiteri, Robert S. Wilson, Francine Grodstein, Julie A. Schneider, Hans-Ulrich Klein, Philip L. De Jager, David A. Bennett

CONTENTS		PAGES
**eTable 1**	Associations of Cortical Peptides Examined in this Study and Motor Decline	2-6
**eTable 2**	Indices of Brain Pathologies and Motor Decline	7
**eTable 3**	Factor Analysis and Factor Functions for Motor Resilience Peptides	8-9
**Supplementary Figure 1**	Motor Resilience Factors and Motor Decline	10
**References**		11-12

eTable 1. Associations of Cortical Peptides Examined in this Study and Motor Decline

	Peptide.Name	Peptide Sequence	Estimate	Standard Error	Raw_p	FDR_p
1	AK4_1	IAQNFGLQHLSSGHFLR	-0.01146	0.00416	0.00589	0.05118
2	AK4_2	SLLVPDHVITR	-0.01002	0.00434	0.02111	0.12893
3	AMPD2_2	HLEEIVHVEQGR	-0.00905	0.0032	0.00466	0.04525
4	ANKRD40_2	GEMPVQLTSR	0.00108	0.0018	0.54695	0.76777
5	AP2A2_1	FFQPTEMASQDFFQR	0.0029	0.00518	0.57648	0.77091
6	AP2A2_2	YGGTFQNVSVQLPITLNK	0.00117	0.00541	0.82929	0.92647
7	AP2B1_2	LHDINAQMVEDQGFLDSLR	-0.00146	0.00392	0.7088	0.88016
8	AP2M1_2	TFITQQGIK	0.00492	0.00565	0.38452	0.66848
9	APOD_1	C[+57]PNPPVQENFDVNK	-0.00232	0.00163	0.15468	0.3719
10	APOD_2	NPNLPPETVDSLK	-0.00249	0.00159	0.11747	0.34036
11	APOE_1	GEVQAMLGQSTEELR	4.28E-04	0.00198	0.8287	0.92647
12	APOE_2	AATVGSLAGQPLQER	0.00602	0.0021	0.0041	0.04525
13	APP_1	EVC[+57]SEQAETGPC[+57]R	-0.00346	0.00167	0.0382	0.1666
14	APP_2	THPHFVIPYR	-0.00414	0.00325	0.20187	0.45171
15	ATP5F1_1	QASIQHIQNAIDTEK	0.0034	0.00451	0.45172	0.72921
16	ATP5F1_2	HVVQSISTQQEK	-0.00542	0.00336	0.1075	0.32392
17	ATP5J2_1	DFSPSGIFGAFQR	0.00271	0.00265	0.3067	0.57907
18	ATP5J2_2	LGELPSWILMR	5.97E-04	0.00386	0.87714	0.93506
19	bA	LVFFAEDVGSNK	-0.00104	2.92E-04	3.70E-04	0.01065
20	bA38	GAIIGLMVGG	6.26E-04	6.98E-04	0.36988	0.65121
21	BCL2L1_1	ELVVDFLSYK	-0.00305	0.00403	0.44875	0.72921
22	BCL2L1_2	EAGDEFELR	0.00198	0.00249	0.4275	0.71041
23	BCR_2	SVGDIEQELER	-0.00993	0.00433	0.02189	0.12975
24	BIN1_1	SPSPPDGSPAATPEIR	-3.10E-04	0.00118	0.79444	0.91691
25	BIN1_2	VNHEPEPAGGATPGATLPK	-9.10E-04	0.00187	0.6245	0.81114
26	BIN1_3	LQAHLVAQTNLLR	0.00564	0.00412	0.17146	0.39949
27	BIN1_4	NQAEEELIK	0.00789	0.00512	0.12356	0.34441
28	BIN1_5	AAPQWC[+57]QGK	2.64E-04	0.00328	0.93581	0.96487
29	BIN1_6	AEEELIK	-6.30E-04	9.13E-04	0.49051	0.75412
30	BIN1_7	AQPSDNAPAK	-0.00183	0.0013	0.15914	0.37465
31	BIN1_9	GPPVPPPPK	0.00312	0.00382	0.41391	0.69515
32	C1QA_1	SLGFC[+57]DTTNK	-6.50E-04	0.00167	0.69696	0.87024
33	C1QA_2	DQPRPAFSAIR	-4.20E-04	0.00164	0.79519	0.91691
34	C9orf16_1	LEFQQQLGEAPSDASP	-0.00153	0.0025	0.54128	0.76607
35	C9orf16_2	LQELLESNR	-0.00393	0.00233	0.0912	0.29028
36	CASS4_2	GTFPLDEDVSYK	0.00104	0.0018	0.56253	0.77026
37	CBX1_1	C[+57]PQVVISFYEER	-0.00349	0.00344	0.31003	0.57907
38	CBX1_2	NSDEADLVPAK	-0.00495	0.00276	0.07261	0.26372
39	CD2AP_1	VLFEYIPQNEDELELK	-0.0038	0.00235	0.10623	0.32392
40	CD44_1	YGFIEGHVVIPR	-0.0023	8.07E-04	0.00445	0.04525
41	CD44_2	ALSIGFETC[+57]R	-0.00235	8.34E-04	0.00481	0.04525
42	CD47_1	STVPTDFSSAK	0.01043	0.00675	0.12195	0.34441
43	CD47_2	EGETIIELK	0.00292	0.00506	0.56445	0.77026
44	CELF1_1	MFVGQVPR	0.00324	0.00551	0.55644	0.77026
45	CLINT1_1	HIHITQATETTTTR	6.01E-04	0.00355	0.86526	0.93095

	Peptide.Name	Peptide Sequence	Estimate	Standard Error	Raw_p	FDR_p
46	CLTA_1	ELEEWYAR	3.01E-04	0.00556	0.95679	0.97504
47	CLU_1	ASSIIDELFQDR	-0.00414	0.00209	0.04782	0.18961
48	CLU_2	LFDSDPITVTVPVEVSR	-0.00559	0.00263	0.0336	0.16211
49	COX7B_2	TPDFHDK	-0.00182	0.00178	0.30436	0.57907
50	CPLX2_1	AALEQPC[+57]EGSLTRPK	0.00195	0.00278	0.48259	0.74703
51	CPLX2_2	YLPGPLQDMFK	-4.70E-04	0.00286	0.86916	0.93095
52	CYC1_1	GLLSSLDHTSIR	0.00581	0.00369	0.11488	0.33804
53	CYC1_2	LFDYFPKPYPNSEAAR	0.01038	0.00455	0.02239	0.12975
54	DIP2B_1	LLSPYSPQTQETDSAVQK	9.95E-04	0.00252	0.69282	0.86988
55	DOCK2_1	DILLPVITK	-0.00147	8.33E-04	0.0781	0.27153
56	ELMO1_1	VAIEWPGAYPK	-7.50E-04	0.0011	0.49794	0.75526
57	ELMO1_2	LLDLENIQIPDAPPPIPK	9.57E-04	0.00212	0.65184	0.82762
58	EPS15_2	SATSSSVSNVVITK	-0.00384	0.00181	0.03371	0.16211
59	EPS15L1_2	GTPGPDSSGSLGSGEFTGVK	0.00626	0.00379	0.09799	0.30759
60	FBXO2_1	VLAALPAAELVQAC[+57]R	-0.00517	0.00247	0.03623	0.1666
61	FBXO2_2	SDAGC[+57]LYELTVK	-0.01049	0.00464	0.02381	0.13451
62	FCER1G_1	SDGVYTGLSTR	3.15E-04	0.00163	0.847	0.93095
63	FCHO2_1	SASNYSQLGTFAPVWDVFK	-0.00184	0.00214	0.38928	0.67159
64	FERMT2_1	LLIPVAEGMNEIWLR	-0.00473	0.00327	0.14796	0.36536
65	FERMT2_2	INQGWLDSSR	-0.00494	0.00314	0.11517	0.33804
66	FYN_1	AQFETLQQLVQHYSER	-0.00691	0.00464	0.13678	0.36505
67	FYN_3	LIEDNEYTAR	-0.02719	0.00587	0	2.10E-04
68	GFAP_1	DNLAQDLATVR	-0.00403	0.0012	7.70E-04	0.01441
69	GFAP_2	ALAAELNQLR	-0.00395	0.00114	5.70E-04	0.01344
70	GRB2_1	FNSLNELVDYHR	-0.0051	0.00411	0.21389	0.4648
71	GRB2_2	VLNEEC[+57]DQNWYK	-0.01864	0.00557	8.30E-04	0.01441
72	GSTP1_1	FQDGDLTLYQSNTILR	-0.00868	0.00294	0.00314	0.0394
73	HLA_B_2	DGEDQTQDTELVETRPAGDR	-0.00103	0.00158	0.51322	0.76308
74	HLA_B_5	WAAVVVPSGEEQR	-0.00194	0.00154	0.20866	0.45785
75	HSPA8_1	NSLESYAFNMK	0.00751	0.00656	0.25228	0.52307
76	HSPA8_2	DAGTIAGLNVLR	0.0106	0.00715	0.13848	0.36505
77	HSPB2_2	TYVLPADVDPWR	-0.00607	0.00181	8.00E-04	0.01441
78	IGFBP5_1	FVGGAENTAHPR	-0.00728	0.00146	0	5.00E-05
79	INPPL1_4	LLLDTLQLSK	-0.00443	0.00262	0.09017	0.29028
80	ITPK1_1	LLAEPAGGLVGER	0.00361	0.00309	0.24217	0.50677
81	KIF5B_2	VFQSSTSQEQVYNDC[+57]AK	0.00183	0.00249	0.46342	0.7324
82	LAMP2_2	GILTVDELLAIR	-0.00265	0.00171	0.12067	0.34441
83	LDHA_1	DQLIYNLLK	0.00898	0.00427	0.03549	0.1666
84	LDHA_2	GEMMDLQHGSLFLR	0.01251	0.00448	0.00525	0.04742
85	LDHB_1	LIAPVAEEEATVPNNK	-0.01125	0.00542	0.03784	0.1666
86	LDHB_2	GEMMDLQHGSLFLQTPK	-0.00717	0.00439	0.10199	0.31576
87	MEF2C_1	NSPGLLVSPGNLNK	0.00222	0.00189	0.23883	0.50444
88	MLF2_2	LAIQGPEDSPSR	-0.00137	0.00287	0.63325	0.81315
89	MTOR_1	VLGLLGALDPYK	-0.00575	0.00565	0.30865	0.57907
90	NCL_1	FGYVDFESAEDLEK	-0.00559	0.00385	0.14642	0.36536
91	NCL_2	GFGFVDFNSEEDAK	-0.00304	0.00373	0.41524	0.69515
92	NDUFA10_1	ITSAYLQDIENAYK	0.00536	0.00243	0.02719	0.1463
93	NDUFA10_2	C[+57]EVLQYSAR	0.01106	0.0042	0.00848	0.06846

	Peptide.Name	Peptide Sequence	Estimate	Standard Error	Raw_p	FDR_p
94	NDUFA5_1	ILDVLEEIPK	0.01076	0.00418	0.01003	0.07559
95	NDUFA5_2	LWEPLVEEPPADQWK	0.00724	0.00365	0.04728	0.18961
96	NDUFA6_2	FFHETEAPRPK	0.01315	0.00414	0.0015	0.02256
97	NDUFA7_1	ALVSGKPAESSAVAATEK	0.01276	0.00405	0.00163	0.02303
98	NDUFA7_2	NWASGHDLQGK	0.00785	0.0035	0.02508	0.13824
99	NDUFS6_1	VIAC[+57]DGGGGALGHPK	0.0054	0.00313	0.08469	0.28153
100	NDUFS6_2	TGTC[+57]GYC[+57]GLQFR	0.00851	0.00356	0.01696	0.11276
101	NDUFV1_1	GPDWILGEIK	0.00925	0.00392	0.01821	0.11756
102	NDUFV1_2	LLEGC[+57]LVGGR	0.00512	0.00351	0.14438	0.36536
103	PADI2_1	ILSNESLVQENLYFQR	-0.00412	0.00162	0.0111	0.07841
104	PADI2_2	ELGLTEQDIIDLPALFK	-0.0038	0.00162	0.01897	0.11907
105	PICALM_1	ITAAQHSVTGSAVSK	6.46E-04	0.00242	0.78948	0.91691
106	PICALM_2	ATTHEIMGPK	6.12E-04	0.00186	0.7419	0.90145
107	PIK3R1_1	FSAASSDNTENLIK	-0.0017	0.00369	0.64465	0.82312
108	PLP1_1	TSASIGSLC[+57]ADAR	-7.00E-05	6.93E-04	0.91857	0.96109
109	PLP1_2	LIETYFSK	-8.00E-05	7.03E-04	0.90736	0.95378
110	PLXNB1_1	GQNLDVVQTPR	-0.00489	0.00236	0.03833	0.1666
111	PLXNB1_2	RPTVEQGLGQLSNLLNSK	-0.00753	0.00267	0.0048	0.04525
112	PTK2B_1	LIGSVDDLLPSLPSSSR	0.0027	0.0043	0.53076	0.76607
113	PTMS_1	SVEAAAELSAK	-0.00993	0.00324	0.00217	0.0288
114	PTPN11_2	IQNTGDYYDLYGGEK	-4.30E-04	0.00455	0.9243	0.96264
115	PTPRD_2	HNVADSQITTIGNLVPQK	-0.00312	0.00318	0.32714	0.60108
116	PVALB_1	FFQMVGLK	0.00104	0.0017	0.54235	0.76607
117	PVALB_2	SGFIEEDELGFILK	0.00113	0.00176	0.52081	0.76576
118	RPL10A_2	ILGPGLNK	0.00701	0.00641	0.27417	0.5633
119	RPL17_1	GLDVDSLVIEHIQVNK	-0.00164	0.0023	0.4756	0.74129
120	RPL35_1	VLTVINQTQK	2.58E-04	0.00106	0.80726	0.92609
121	RPL35_2	YKPLDLRPK	0.0059	0.0027	0.02923	0.15361
122	RPL35A_1	DETEFYLGK	6.46E-04	0.00379	0.86462	0.93095
123	RPL35A_2	VMLYPSR	0.00223	0.00248	0.36798	0.65121
124	RPS2_1	SPYQEFTDHLVK	0.00615	0.00357	0.08471	0.28153
125	RPS2_2	TYSYLTPDLWK	0.00456	0.00455	0.31618	0.5857
126	RUVBL1_1	TALALAIAQELGSK	-0.00652	0.00452	0.14873	0.36536
127	RUVBL1_2	YSVQLLTPANLLAK	-0.01049	0.00491	0.03253	0.16211
128	SASH1_1	TC[+57]SFGGFDLTNR	-0.00204	0.00189	0.27914	0.56833
129	SASH1_2	LLVDSQGLSGC[+57]SPR	-0.00156	0.00171	0.36191	0.64914
130	SDHA_2	VGSVLQEGC[+57]GK	-0.00216	0.00417	0.60543	0.79091
131	SDHB_1	YLGPAVLMQAYR	0.00258	0.00388	0.50637	0.75813
132	SDHB_2	DLVPDLSNFYAQYK	0.0025	0.00444	0.57258	0.77026
133	SDHC_1	SLC[+57]LGPALIHTAK	6.61E-04	0.00213	0.75588	0.90386
134	SDHC_2	HLMWDLGK	1.16E-04	0.00219	0.95778	0.97504
135	SDHD_1	AASLHWTSER	-0.00201	0.00327	0.53878	0.76607
136	SDHD_2	AVAMLWK	-7.90E-04	0.00237	0.73879	0.90145
137	SLC6A12_1	QLITPDSSLPQPK	0.00169	0.00263	0.5218	0.76576
138	SNAP25_1	HMALDMGNEIDTQNR	0.00832	0.00327	0.01108	0.07841
139	SNAP25_2	AWGNNQDGVVASQPAR	0.0075	0.00382	0.04977	0.19391
140	SNAP25_3	ADQLADESLESTR	0.00357	0.00297	0.22887	0.48797
141	SNAP25_4	TLVMLDEQGEQLER	0.00512	0.00295	0.08329	0.28153

	Peptide.Name	Peptide Sequence	Estimate	Standard Error	Raw_p	FDR_p
142	SNAP25_6	FC[+57]GLC[+57]VC[+57]PC[+57]NK	0.00172	0.00234	0.46322	0.7324
143	SNAP25_7	C[+57]C[+57]GLFIC[+57]PC[+57]NK	0.00326	0.00231	0.1583	0.37465
144	SNAP29_1	DLPDGPDAPADR	-0.00865	0.00436	0.04734	0.18961
145	SNAP29_2	SVFGGLVNYFK	0.00491	0.00549	0.37171	0.65121
146	SNAP47_1	NVVFSIIEHFWR	-0.0019	0.00335	0.56951	0.77026
147	SNCA_1	EGVVHGVATVAEK	-1.00E-04	0.00376	0.97896	0.97896
148	SNCA_2	TVEGAGSIAAATGFVK	7.47E-04	0.00337	0.82429	0.92647
149	SPP1_1	AIPVAQDLNAPSDWDSR	-0.00184	8.63E-04	0.03353	0.16211
150	STX12_1	LMNDFSAALNNFQAVQR	-2.40E-04	0.00539	0.96429	0.97551
151	STX12_2	ELGSLPLPLSTSEQR	0.00255	0.00888	0.77371	0.91691
152	STX16_1	IDYNVEQSC[+57]IK	0.00273	0.00293	0.35024	0.63323
153	STX16_2	LTDAFLLLR	0.00289	0.00239	0.22715	0.48797
154	STX18_1	TAVLDFIEDYLK	-6.30E-04	0.0023	0.78532	0.91691
155	STX18_2	TC[+57]SEAIQQLR	-7.80E-04	0.00368	0.83218	0.92647
156	STX1A_1	SIEQSIEQEEGLNR	0.01346	0.00378	3.80E-04	0.01065
157	STX1A_2	QALSEIETR	0.0093	0.00328	0.00464	0.04525
158	STX1B_1	QHSAILAAPNPDEK	0.00334	0.00409	0.41415	0.69515
159	STX1B_2	AIEQSIEQEEGLNR	0.00388	0.00415	0.34911	0.63323
160	STX4_1	NILSSADYVER	-1.00E-04	0.00268	0.97119	0.97551
161	STX4_2	HSEIQQLER	2.02E-04	0.00261	0.93823	0.96487
162	STX5_1	HLQTHSNTIVVSLQSK	2.16E-04	0.00154	0.8883	0.93811
163	STX7_1	TLNQLGTPQDSPELR	-0.00197	0.00849	0.8165	0.92647
164	STX7_2	EFGSLPTTPSEQR	-0.00364	0.00696	0.60044	0.79091
165	STXBP1_1	SSASFSTTAVSAR	0.00476	0.00467	0.30819	0.57907
166	STXBP1_2	HIAEVSQEVTR	0.0043	0.0042	0.30636	0.57907
167	STXBP1_3	WEVLIGSTHILTPQK	-0.00138	0.00408	0.7352	0.90145
168	STXBP1_4	WEVLIGSTHILTPTK	0.00362	0.00286	0.20615	0.45675
169	STXBP1_5	TDEEISS	-0.00471	0.00173	0.00646	0.05405
170	STXBP1_6	VSFEDQAPTME	0.00927	0.0027	5.90E-04	0.01344
171	STXBP3_2	AAYIYFTDFC[+57]PDNLFNK	-0.00133	0.00254	0.59927	0.79091
172	STXBP5_2	LLQPVIVSPSGTILR	-0.00505	0.00388	0.19373	0.44225
173	STXBP6_2	QFEGSTSFVR	-0.0031	0.00295	0.29362	0.57907
174	SV2A_1	FEEEDDDDFPAPSDGYYR	5.53E-04	0.00233	0.81202	0.92647
175	SV2A_2	GGLSDGEGPPGGR	-0.0013	0.00221	0.55534	0.77026
176	SV2B_1	ATAFGILNGLC [+57] K	7.19E-04	0.00252	0.77497	0.91691
177	SV2B_2	VFTVSNIK	3.39E-04	0.00241	0.88781	0.93811
178	SYN1_1	QGPPQKPPGPAGPTR	-0.00393	0.00256	0.12496	0.34441
179	SYN1_2	LGTEEFPLIDQTFYPNHK	-0.00279	0.0027	0.30183	0.57907
180	SYN2_1	SFRPDFVLIR	-0.00436	0.00244	0.07352	0.26372
181	SYN2_2	VLLVVDEPHADWAK	-0.00513	0.0028	0.06693	0.24795
182	SYN3_1	SFKPDFILVR	6.36E-04	0.00341	0.85187	0.93095
183	SYN3_2	SQSLTNSLSTSDTSQR	-5.50E-04	0.00298	0.85327	0.93095
184	SYT1_1	VFVGYNSTGAELR	0.00373	0.00247	0.13141	0.35782
185	SYT1_2	LTVVILEAK	0.00146	0.00238	0.5399	0.76607
186	SYT11_1	NLLVDAAEAGLLSR	0.00124	0.00397	0.7554	0.90386
187	SYT12_1	VSLLPDEQIVGISR	0.00728	0.0022	9.60E-04	0.01546
188	SYT2_1	IFVGSNATGTELR	-9.00E-04	0.00131	0.49408	0.75447
189	SYT2_2	LTVC[+57]ILEAK	-6.10E-04	0.00138	0.65759	0.83026

	Peptide.Name	Peptide Sequence	Estimate	Standard Error	Raw_p	FDR_p
**190**	SYT7_1	NLNPIFNESFAFDIPTEK	0.00359	0.00335	0.28319	0.57143
**191**	SYT7_2	NSLETVGTPDSGR	0.00597	0.00294	0.04238	0.18072
**192**	TARDBP_1	FGGNPGGFGNQGGFGNSR	-0.00317	0.00477	0.50654	0.75813
**193**	TARDBP_2	TSDLIVLGLPWK	-0.00487	0.00632	0.44158	0.72316
**194**	tau_12E8	IGSTENLK	-0.00399	0.00209	0.05569	0.21331
**195**	tau_12E8_s262	IGS[+80]TENLK	-0.00226	5.27E-04	2.00E-05	8.00E-04
**196**	tau_77G7	HVPGGGSVQIVYKPVDLSK	-7.90E-04	0.00245	0.74684	0.9026
**197**	tau_77G7_s305	HVPGGGS[+80]VQIVYKPVDLSK	-5.30E-04	6.85E-04	0.43694	0.72079
**198**	tau_AT100	TPSLPTPPTREPK	0.00369	0.0059	0.53173	0.76607
**199**	tau_AT100_t217	TPSLPT[+80]PPTREPK	-0.00409	0.00106	1.20E-04	0.00458
**200**	tau_AT8	SGYSSPGSPGTPGSR	0.00447	0.00312	0.15171	0.36866
**201**	tau_AT8_s202	SGYSSPGS[+80]PGTPGSR	-0.0024	0.00182	0.18609	0.42914
**202**	tau_PHF1	SPVVSGDTSPR	-3.20E-04	0.00192	0.86865	0.93095
**203**	tau_PHF1_s404	SPVVSGDTS[+80]PR	-0.00258	0.00136	0.05828	0.21954
**204**	TPRG1L_1	TASLC[+57]QLESFK	-0.00744	0.00286	0.0093	0.07245
**205**	TRIM28_1	LSPPYSSPQEFAQDVGR	-0.00369	0.00453	0.41523	0.69515
**206**	TRIM28_2	VFPGSTTEDYNLIVIER	-6.00E-04	0.00313	0.84745	0.93095
**207**	UGT8_2	LIEWLPQNDLLGHSK	-4.30E-04	8.90E-04	0.63048	0.81315
**208**	UNC5C_1	IAC[+57]TTLC[+57]PVDGR	-4.90E-04	0.00176	0.78025	0.91691
**209**	UQCR10_1	AFDQGADAIYDHINEGK	7.57E-04	0.00146	0.60262	0.79091
**210**	UQCR10_2	LYSLLFR	0.00249	0.00439	0.57014	0.77026
**211**	UQCRC2_1	TIAQGNLSNTDVQAAK	0.00772	0.00436	0.07702	0.27153
**212**	UQCRC2_2	YEDFSNLGTTHLLR	0.00447	0.00424	0.29137	0.57907
**213**	VAMP1_1	ADALQAGASQFESSAAK	-0.00138	0.00193	0.47391	0.74129
**214**	VAMP1_2	LQQTQAQVEEVVDIIR	8.40E-05	0.0021	0.96805	0.97551
**215**	VAMP5_1	SDQLLDMSSTFNK	-0.00375	0.00189	0.04678	0.18961
**216**	VAPB_1	EAKPEDLMDSK	0.00758	0.00514	0.14033	0.36505
**217**	VAPB_2	VEQVLSLEPQHELK	0.01584	0.00656	0.0157	0.1075
**218**	VAT1_1	GVDIVMDPLGGSDTAK	-0.00339	0.00198	0.08629	0.28262
**219**	VAT1_2	TWWNQFSVTALQLLQANR	-0.00237	0.00186	0.20165	0.45171
**220**	VDAC2_1	VNNSSLIGVGYTQTLRPGVK	-2.40E-04	0.0031	0.93926	0.96487
**221**	VDAC2_2	LTLSALVDGK	-0.00108	0.00304	0.72225	0.89196
**222**	VGF_1	LADLASDLLLQYLLQGGAR	0.00722	0.00139	0	2.00E-05
**223**	VGF_2	LQEQEELENYIEHVLLR	0.00801	0.00151	0	2.00E-05
**224**	VPS35_2	FTLPPLVFAAYQLAFR	-0.00448	0.00304	0.14053	0.36505
**225**	VSNL1_1	SDPSIVLLLQC[+57]DIQK	0.00696	0.00482	0.14859	0.36536
**226**	VSNL1_2	LNLEEFQQLYVK	0.00448	0.00603	0.45723	0.7324

In 226 separate linear mixed effects models, the association of each of the 226 peptides was examined with trajectory of motor function prior to death. Each model includes seven terms: Time (the annual rate of change in motor decline prior to death), as well as age, sex and each peptide and their interaction with Time. Each row shows the results for the interaction of Time with the peptide shown in the left column; Estimate, Standard Error and raw and FDR p-Value; (results for the other terms are not shown). In peptide sequence modifications, cysteine carbamidomethylation and phosphorylation are denoted as [+57] and [+80], respectively.

eTable 2. Indices of Brain Pathologies and Motor Decline

Model Term	Estimate	Standard Error, p-Value
Time	-0.024	0.002, <0.001
Age at death	-0.011	0.001, <0.001
Sex	0.047	0.018, 0.010
AD pathology	-0.028	0.015, 0.052
Lewy bodies	-0.035	0.021, 0.095
Nigral neuronal loss	-0.042	0.012, <0.001
TDP-43	0.005	0.008, 0.501
Hippocampal sclerosis	0.011	0.031, 0.729
Macroinfarct	-0.074	0.018, <0.001
Microinfarct	-0.027	0.019, 0.147
Atherosclerosis	-0.029	0.011, 0.008
Arteriolosclerosis	-0.026	0.010, 0.008
CAA	-0.012	0.008, 0.147
Time *Age at death	0.0005	0.0002, <0.001
Time*Sex	-0.004	0.002, 0.040
Time *AD pathology	**-0.004**	**0.002, 0.009**
Time *Lewy bodies	-0.004	0.002, 0.050
Time *Nigral neuronal loss	**-0.004**	**0.001, 0.002**
Time *TDP-43	-0.0000	0.001, 0.965
Time *Hippocampal sclerosis	0.002	0.003, 0.457
Time *Macroinfarct	**-0.004**	**0.002, 0.034**
Time *Microinfarct	0.0001	0.002, 0.938
Time *Atherosclerosis	**-0.003**	**0.001, 0.004**
Time *Arteriolosclerosis	-0.001	0.001, 0.193
Time *CAA	-0.001	0.001, 0.103

This table shows the results for a single linear mixed-effects model that examines the association of each of its terms with the level of motor function proximate to death and the annual rate of change in motor decline prior to death. This model includes twenty-five terms including: Time, the annual rate of change in motor decline prior to death, as well as age, sex and 10 indices of brain pathology and their interaction with Time. Each row shows the Estimate, Standard Error and p-Value for the single term shown in the left column.

eTable 3. Factor Analysis and Factor Functions for Motor Resilience Peptides

Rotated Factor Pattern of Motor Resilience Peptides					
Peptide	Factor 1	Factor 2	Factor 3	Factor 4	Factor 5
	Cell Structure	Signal Transduction	Synaptic Plasticity	Cell Proliferation	APOE
**GFAP_2**	0.922	-0.216	-0.203	0.051	-0.013
**GFAP_1**	0.913	-0.223	-0.184	0.058	0.020
**CD44_2**	0.829	-0.343	-0.276	-0.199	-0.025
**CD44_1**	0.822	-0.339	-0.283	-0.197	-0.007
**GSTP1_1**	0.519	-0.432	-0.438	-0.245	-0.023
**NDUFA7_1**	-0.295	0.757	0.227	0.192	-0.001
**NDUFA6_2**	-0.310	0.754	0.220	0.155	-0.001
**STXBP1_6**	-0.197	0.739	0.078	-0.028	-0.173
**FYN_3**	0.260	-0.461	-0.23	-0.031	-0.325
**GRB2_2**	0.350	-0.517	-0.239	-0.091	-0.321
**SYT12_1**	-0.319	0.573	0.602	0.196	0.044
**VGF_2**	-0.162	0.271	0.846	-0.053	0.112
**VGF_1**	-0.142	0.292	0.827	-0.068	0.093
**STX1A_1**	-0.307	0.299	0.658	0.209	-0.075
**tau_12E8_s262**	0.145	-0.133	-0.397	0.183	0.342
**HSPB2_2**	0.195	0.114	-0.468	0.095	0.100
**PTMS_1**	-0.030	0.261	0.173	0.770	0.024
**IGFBP5_1**	0.023	-0.401	-0.269	0.654	0.086
**LDHA_2**	0.184	-0.316	0.133	-0.508	0.021
**APOE_2**	-0.011	-0.018	-0.024	-0.011	0.856

The cells highlighted in yellow in each column in the Table were included in the Factor at the top of the column. Further inspection of the functions of the individual proteins clustering within each factor suggested that each factor might represent distinct biological functions as discussed below.

Factor 1 represents proteins involved in maintenance of the brain's structural integrity. ***GFAP*** (Glial Fibrillary Acidic Protein) is a crucial protein of astrocytes that provides structural and functional support for neurons (1). ***CD44*** is a transmembrane glycoprotein that is involved in diverse cellular processes acting as a bridge between the extracellular matrix and intracellular cytoskeleton (2). ***GSTP1*** protein **(**Glutathione S-Transferase P1) is a member of glutathione transferase family that is involved in detoxification of toxic compounds, and its genetic polymorphism found associated with Parkinson disease and AD (3).

Factor 2 represents proteins involved in mitochondrial bioenergetics and synaptic signaling. Mitochondrial complex I NADH dehydrogenase is the first enzyme complex in the electron transport chain of mitochondria that are involved in many cellular processes including synaptic signaling(6). NDU has 45 subunits, including subunit 7 (***NDUFA7***) and 6 (***NDUFA6***), and its level is reportedly lower in AD and PD patients (7). ***STXBP1*** (Syntaxin binding protein 1) is a protein involved in synaptic vesicle fusion machinery, and its gene mutation is associated with parkinsonism (4). ***SYT12*** (Synaptotagmin 12) is a member of a group of proteins that are crucial synaptic vesicle (5). ***GRB2*** protein (Growth factor receptor bound protein 2) is involved in regulation of signaling events (8), and its level may be higher in AD brains (9).

Factor 3 represents proteins involved in synaptic plasticity. ***STX1A*** *protein* (Syntaxin1A) is a member of the SNARE complex that is involved in intracellular vesicular trafficking including fusion of vesicles with synaptic membrane, and ***SYT12*** is also one of the SNARE regulatory proteins(10). ***VGF*** nerve growth factor inducible, is a peptide precursor whose expression is easily inducible by nerve growth factors which is divided by proteolysis into polypeptides of different mass. It has diverse physiological roles including enhancement of synaptic plasticity, neurogenesis and energy homeostasis (11).

Factor 4 represents proteins involved in cell proliferation. ***PTMS*** (parathymosin) is a small acidic nuclear protein that is involved in cell cycle progression and cell proliferation (12), and its level is associated with microglial activation induced by amyloid-beta plaques (13). ***IGFBP5*** (Insulin like growth factor binding protein 5) is a regulatory protein with different biological actions including inhibition of insulin-like growth factor signaling(14), and has been reported to be associated with poor motor and cognitive function in late-life (15). ***LDHA*** (Lactate Dehydrogenase A) is a protein that catalyzes conversion of L-lactate and NAD into pyruvate and NADH in the final step of anaerobic glycolysis. Proliferating cells, rely on this metabolic pathway, and have high expression levels of LDHA gene (16). *LDHA* gene expression has been reported to be downregulated in AD (17).

Factor 5 was comprised of only one protein, ***APOE*** (Apolipoprotein E). The APOE gene is the best know genetic risk factor for late-life onset AD. Its ε4 allele is associated with increased risk of AD dementia and risk of poor motor function while its ε2 allele is associated with improved function (18)**.**

Supplementary Figure 1. Motor Resilience Factors and Motor Decline.

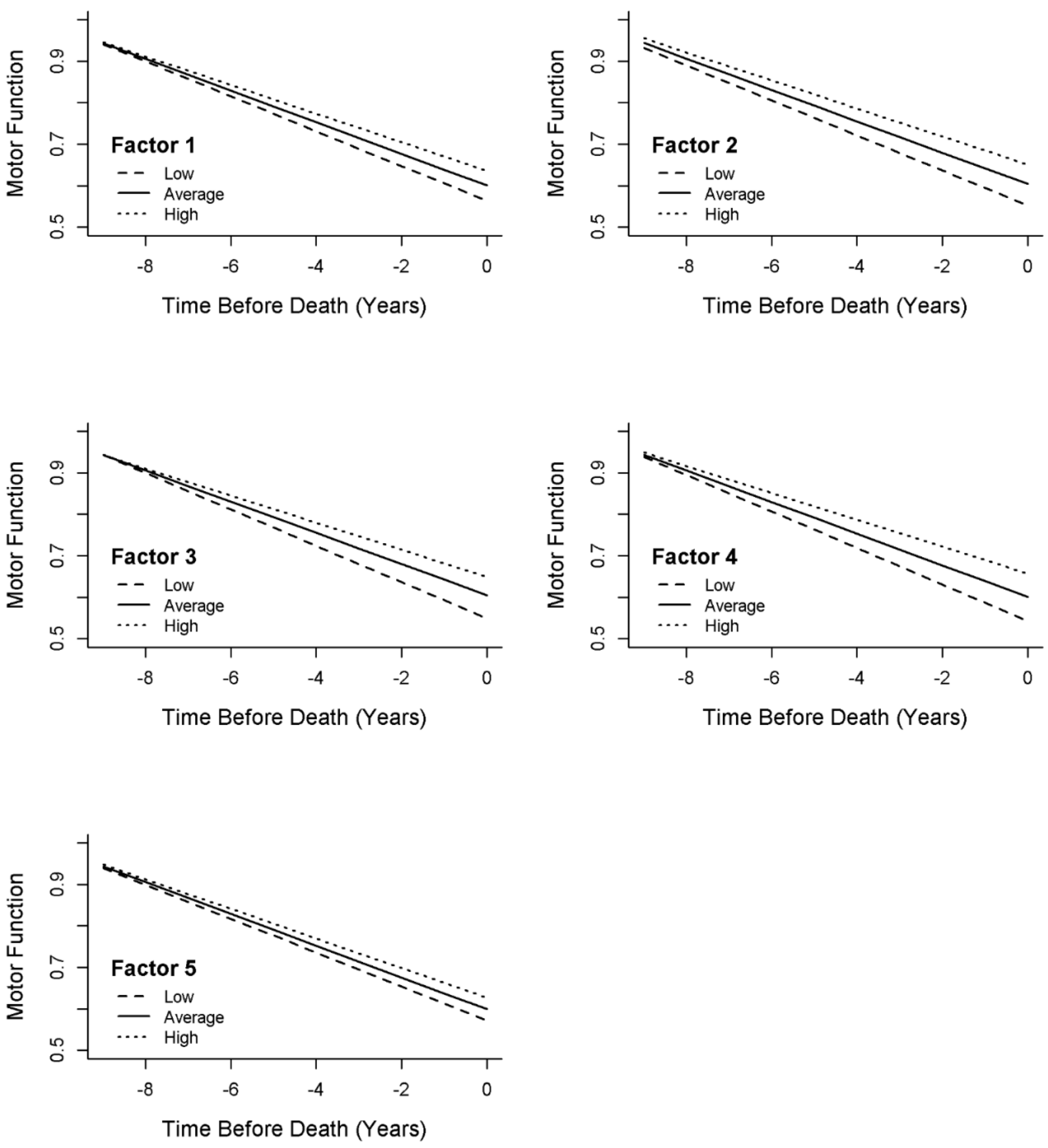

We constructed an average score for each of the 4 factors based on the constituent peptides obtained in the factor analysis (**Figure 4b; Table 2**). Factor 5 was based on the expression level of APOE. The figure illustrates the model derived trajectory of motor decline for three average older adults with high (90th percentile), average (50th percentile) and low (10th percentile) levels of each of the five factors.

REFERENCES

1. Yang Z, Wang KKW. Glial fibrillary acidic protein: from intermediate filament assembly and gliosis to neurobiomarker. Trends Neurosci. 2015 Jun;38(6):364–74.
2. Ponta H, Sherman L, Herrlich PA. CD44: From adhesion molecules to signalling regulators. Nat Rev Mol Cell Biol. 2003 Jan;4(1):33–45.
3. Bocedi A, Noce A, Marrone G, Noce G, Cattani G, Gambardella G, et al. Glutathione Transferase P1-1 an Enzyme Useful in Biomedicine and as Biomarker in Clinical Practice and in Environmental Pollution. Nutrients. 2019 Jul 27;11(8):1741.
4. Lanoue V, Chai YJ, Brouillet JZ, Weckhuysen S, Palmer EE, Collins BM, et al. *STXBP1* encephalopathy: Connecting neurodevelopmental disorders with α-synucleinopathies? Neurology. 2019 Jul 16;93(3):114–23.
5. Jahn R, Fasshauer D. Molecular machines governing exocytosis of synaptic vesicles. Nature. 2012 Oct;490(7419):201–7.
6. Abramov AY, Angelova PR. Cellular mechanisms of complex I-associated pathology. Biochem Soc Trans. 2019 Nov 26;BST20191042.
7. Holper L, Ben-Shachar D, Mann J. Multivariate meta-analyses of mitochondrial complex I and IV in major depressive disorder, bipolar disorder, schizophrenia, Alzheimer disease, and Parkinson disease. Neuropsychopharmacology. 2019 Apr;44(5):837–49.
8. Lee S-E, Jeong S, Lee U, Chang S. SGIP1α functions as a selective endocytic adaptor for the internalization of synaptotagmin 1 at synapses. Mol Brain. 2019 Dec;12(1):41.
9. Majumder P, Roy K, Singh BK, Jana NR, Mukhopadhyay D. Cellular levels of Grb2 and cytoskeleton stability are correlated in a neurodegenerative scenario. Dis Model Mech. 2017 May 1;10(5):655–69.
10. Cupertino RB, Kappel DB, Bandeira CE, Schuch JB, da Silva BS, Müller D, et al. SNARE complex in developmental psychiatry: neurotransmitter exocytosis and beyond. J Neural Transm. 2016 Aug;123(8):867–83.
11. Lewis JE, Brameld JM, Jethwa PH. Neuroendocrine Role for VGF. Front Endocrinol [Internet]. 2015 Feb 2 [cited 2020 Jun 8];6. Available from: http://journal.frontiersin.org/Article/10.3389/fendo.2015.00003/abstract

12. Vareli K, Frangou-Lazaridis M, van der Kraan I, Tsolas O, van Driel R. Nuclear Distribution of Prothymosin α and Parathymosin: Evidence That Prothymosin α Is Associated with RNA Synthesis Processing and Parathymosin with Early DNA Replication. Exp Cell Res. 2000 May;257(1):152–61.

13. Walker D. Gene expression profiling of amyloid beta peptide-stimulated human post-mortem brain microglia. Neurobiol Aging. 2001 Dec;22(6):957–66.

14. Duan C, Allard JB. Insulin-Like Growth Factor Binding Protein-5 in Physiology and Disease. Front Endocrinol. 2020 Mar 3;11:100.

15. Buchman AS, Yu L, Petyuk VA, Gaiteri C, Tasaki S, Blizinsky KD, et al. Cognition may link cortical IGFBP5 levels with motor function in older adults. Ginsberg SD, editor. PLOS ONE. 2019 Aug 12;14(8):e0220968.

16. Vander Heiden MG, Cantley LC, Thompson CB. Understanding the Warburg Effect: The Metabolic Requirements of Cell Proliferation. Science. 2009 May 22;324(5930):1029–33.

17. Vallée A, Lecarpentier Y, Guillevin R, Vallée J-N. Reprogramming energetic metabolism in Alzheimer's disease. Life Sci. 2018 Jan;193:141–52.

18. Oveisgharan S, Buchman AS, Yu L, Farfel J, Hachinski V, Gaiteri C, et al. *APOE* ε2ε4 genotype, incident AD and MCI, cognitive decline, and AD pathology in older adults. Neurology. 2018 Jun 12;90(24):e2127–34.
